# Influence of Post-Weld Heat Treatment on the Performance of UHSS Joints

**DOI:** 10.3390/ma18122792

**Published:** 2025-06-13

**Authors:** Mustafa Tümer, Alptekin Kısasöz, Florian Pixner, Norbert Enzinger

**Affiliations:** 1 Welding Technology Program, Kocaeli University, 41001 Kocaeli, Türkiye; mustafa.tumer@kocaeli.edu.tr; 2 Department of Metallurgical and Materials Engineering, Yildiz Technical University, 34220 İstanbul, Türkiye; akisasoz@yildiz.edu.tr; 3 Institute of Materials Science, Joining and Forming, Graz University of Technology, 8010 Graz, Austria; norbert.enzinger@tugraz.at

**Keywords:** EBW, UHSS, microstructure, hardness, toughness

## Abstract

Ultra-high strength steel (UHSS) contributes significantly to lightweight design, environmental compatibility and lower fuel consumption. However, it is essential to maintain excellent mechanical properties in terms of structural integrity, strength and ductility after the applied welding process. In this study, the effect of post-welding heat treatments on the welding of UHSS S1100MC was investigated in order to compensate for the deterioration in toughness that occurred as a result of joining by electron beam welding. Electron beam welding (EBW) provides high energy density and therefore relatively low heat input compared to arc welding. However, the narrow fusion zone (FZ) and heat-affected zone (HAZ) may have insufficient toughness values due to rapid cooling of the joint. In order to protect the relationship between strength and toughness, both the material and the joint were subjected to heat treatment at 500, 650 and 750 °C temperatures for 2 h and were cooled in the furnace. Microstructural characterization and mechanical testing, namely hardness, Charpy impact and tensile tests, were performed to correlate the influence of post-weld heat treatment on the microstructural formation and the corresponding mechanical properties. While the material and the joint maintained their hardness values at 500 °C of around 412 ± 15 HV_0.2_, there was an approximately 8% decrease in hardness to 378 ± 18 HV_0.2_ at 650 °C. At 750 °C, it dramatically lost its high hardness properties, resulting in a low 178 ± 9 HV_0.2_. However, direct quenching from the austenitic temperature resulted in fresh martensite, which provided both the required strength and toughness values in the EBW joint. With a hardness of 437 HV_0.2_, a tensile strength of 1345 MPa and a fracture elongation of more than 9%, superior mechanical properties could be achieved.

## 1. Introduction

The yield strength of steel used in engineering structures gradually increases with the development of high-strength steel materials [1]. The increasing demand for energy efficiency has led to a shift towards structural lightweight [2] technology in production, making it one of the effective measures to enhance the energy efficiency of these high-strength steels [3]. These steels’ high strengths can be achieved through different manufacturing processes. To obtain superior mechanical properties, steels can undergo quenching and tempering (QT) treatments. The relatively high carbon content and quenching with water result in a martensitic microstructure for QT steel, followed by a tempering process. Additionally, microalloying elements (such as Nb, V, and Ti) can be added to further refine the grain size [4,5]. Today, most of the steels produced are joined using different fusion welding methods. It is important to choose the correct welding method and parameters to ensure that the joints provide at least the mechanical properties of the base material [6]. UHSS produced through thermomechanical processing (TMP) provides a wide replacement for steels produced by conventional re-austenitization and quenching. High deformation rates in the non-recrystallizing austenite region provide enhanced refinement during subsequent transformation to martensite, thus offering greater benefits in terms of strength and toughness [7]. Most of the microstructural changes in steel occur at temperatures above 500 °C, so the t_8/5_ guideline has been used as a guide to achieve desired welding quality. The concept of t_8/5_ is still applicable for ultra-high and high-strength steels because it concerns not only the time spent cooling between 800 and 500 °C but also the entire thermal cycle, including the time spent at high temperatures. Longer t_8/5_ times are associated with higher heat input, which means longer periods at temperatures above 1000 °C where austenite grain growth occurs [8]. EBW (Electron Beam Welding) is a special welding process technology that allows thick parts to be welded in a single pass without using filler material [9,10]. The high energy density in the EBW method offers many advantages over traditional arc welding methods, such as high welding speed, deep penetration, narrow fusion and heat affected zone, low heat input and distortion risk [11,12]. However, the formation of fresh martensitic structure in the fusion zone can increase hardness while significantly reducing toughness. Therefore, thermal treatment might become necessary, especially for materials like Ultra-High Strength Steels (UHSS), to improve the different dynamic effects of EBW. Thermal treatment can help enhancing the mechanical properties of the material after welding and mitigate potential hardness issues in the fusion zone [10].

Sisodia and Gáspár [13] investigated that S960QL base material with a thickness of 15 mm were welded without filler material using EBW process. Moreover, the test results of EBW samples were compared with GMAW joining of the same steel grade and thickness. In the applied tensile tests of welded samples, strength values of the as-received tensile strength of S960QL were obtained. The notch impact value of the fusion zone met the requirement of 27 J at −40 °C with an average of 44 J ± 20 for EBW. Steimbreger et al. employed the EBW process to join UHSS plates and applied fatigue tests to determine the strength of the welds under constant amplitude loading. They reported that weld defects (undercuts and underfills) promoted crack nucleation at the toe of the weld for EBW after carrying out the fatigue test. The measured average defect size from metallographic inspection was around 100 µm [14].

In partially lower-strength UHSS (S960QL) materials produced using different manufacturing methods and welded by EBW [13], even when mechanical properties are maintained within desired ranges, rapid cooling, which leads to an increase in yield strength to 1100 MPa, results in excessive hardening tendencies in these steels, causing issues with toughness values. In their previous study [10], the authors found that toughness values remained below 27 J at −40 °C for both standard and notched specimens.

Taking into consideration the latest aforementioned literature, there is a lack of knowledge about post-weld heat treatment of electron beam welded UHSS; more specifically, how heat treatments around austenitizing temperatures affect the mechanical properties and optimization of Charpy impact values of thick-walled electron-beam-welded high-strength steel joints. In particular, this study has the objective of achieving the requested minimum impact toughness of 27 J at −20 °C, in combination with retaining high tensile strength, which is characteristic of the UHSS material class. The present study addresses this subject and represents a novelty in the field by determining the influence of heat treatment parameters on the mechanical properties of S1100MC steel, described by methods such as hardness, impact and tensile tests. The identification of the correlation between heat treatment and mechanical properties is supported by microstructural characterization and provides new insights to improve the mechanical performance of EBW S1100MC and extends the limited literature on EBW UHSS.

## 2. Materials and Methods

### 2.1. Material

The base metal S1100MC (Voestalpine Steel Company, Linz, Austria) with a 20 mm thickness was used in the experimental study. The chemical composition of the base metal consisted of 0.13% C, 1.6% Mn, 0.6% Cr, 0.3% Ni, 0.6% Mo and a total 0.12% of V, Nb and Ti. The S1100MC steel has been widely used due its excellent strength and impact toughness. The UHSS has a minimum yield strength of 1100 MPa and a tensile strength between 1120 and 1300 MPa with a fracture elongation of at least 8% [15].

### 2.2. Welding Procedure and Heat Treatment Conditions

**Figure 1:**
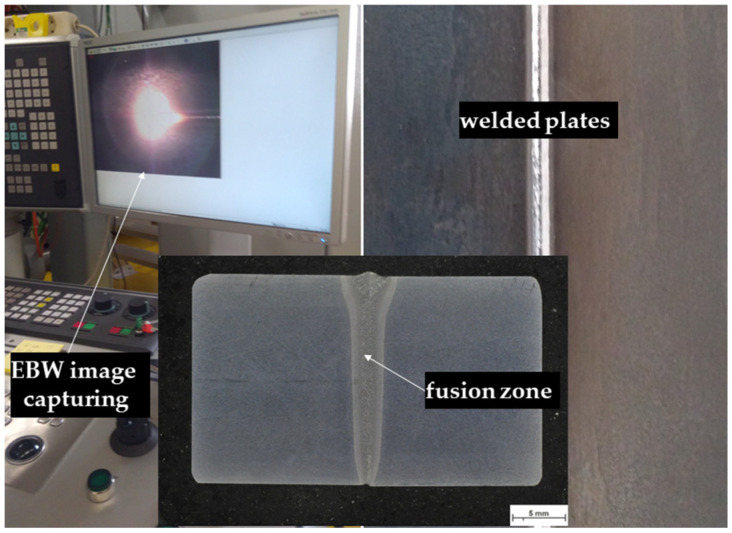
Monitoring system of welding process, welded plates and macrograph.

Joining operations were performed utilizing the Pro-beam EBG 45-150 K14 model electron beam welding device (pro-beam GmbH & Co. KG, Gilching, Germany) without using filler metal. The pressure in the working chamber was maintained at levels below 5 × 10^−3^ mbar. The workpiece was positioned and manipulated using the machine table in the vacuum chamber. The joining position was determined using the electron-optic (ELO) mode and electron beam welding was then carried out in the PA position for the butt joint. Optimized welding parameters were used, which were selected on the basis of a previously conducted parameter study with the aim of achieving complete weld penetration and sound root formation. The optimized welding parameters are as follows: 120 kV acceleration voltage, 63 mA beam current and 12 mm/s welding speed. After welding, heat treatments were applied to improve the microstructure and mechanical properties of the weld. In order to obtain desired microstructure and mechanical properties, heat treatments were carried out in the MSE M-1300 heat treatment furnace (Kocaeli, Türkiye) under the conditions specified in Table 1. Also, the monitoring system of EBW process, welded plates and macrograph are given Figure 1.

Additionally, a phase diagram of the alloy used in the experimental study was calculated using Thermo-Calc 2022 software. The phase diagram was modelled based on the measured chemical composition of the alloy.

### 2.3. Microstructural and Mechanical Characterization

Microstructure examinations of the base metal and weld metal were primarily carried out with Zeiss Axio Scope A1 and Zeiss Axio Observer Inverted (Zeiss, Oberkochen, Germany) light optical microscopes (LOM). The cross-section of the specimens taken from the weldments were ground using sandpapers and polished using diamond pastes with particle sizes of 3 µm and 1 µm. The samples were etched in 3% Nital solution for 15 s for microstructural examinations following the standard grinding and polishing steps. The scanning electron microscopy (SEM) analyses were performed with JEOL JSM-6060LV (Tokyo, Japan).

The mechanical test samples were obtained by mechanical processing methods. Transverse tensile tests of welded joint were performed with a 600 kN load capacity Besmak BMT 600S (Ankara, Türkiye) universal test device according to the EN ISO 6892-1 standard [16]. The test speed was determined as 4 mm/min and the tests were carried out at room temperature. The notch impact tests were performed at −20 °C and the notch was placed at a depth of 1 mm from the top surface of each specimen according to DIN EN ISO 148-1 [17] and ISO 9016 [18]. The specimens were subjected to metallographic procedures to ensure that the notches were positioned precisely within the complete fusion zone. Three samples of each condition were tested to avoid possible unrequested deviation in the impact tests, and mean values and standard deviations were determined. The error bars in the graphs in the results section represent ± standard deviation (SD) from the corresponding mean values. Moreover, the fracture surface of the samples was investigated by SEM analyses. The Vickers hardness measurements were applied with a diamond pyramid indenter under a load of 200 grf, according to ISO 6507-1 [19]. The distances between the HV_0.2_ hardness imprints were 1 mm for the EBW specimen. The dwell time for each imprint was settled as 10 s.

## 3. Results and Discussion

### 3.1. Thermo-Calc Analysis

**Figure 2:**
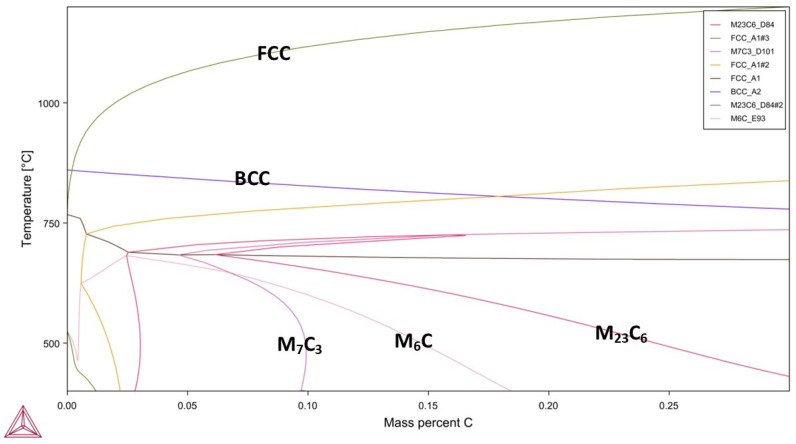
Thermo-Calc analysis of the base metal.

Thermo-Calc analysis of the base metal is given in Figure 2. The analysis exhibits that M_23_C_6_ and M_7_C_3_ carbides are formed below 700 °C. On the other hand, the carbides dissolve above 800–850 °C; in this temperature range the alloy has an austenitic microstructure. Especially considering that the alloy contains Cr, V, Ti and Nb, it can be stated that metal carbides can precipitate at temperatures below 700 °C.

### 3.2. Characterization of the Base Material Before and After the Heat Treatment Process

**Figure 3:**
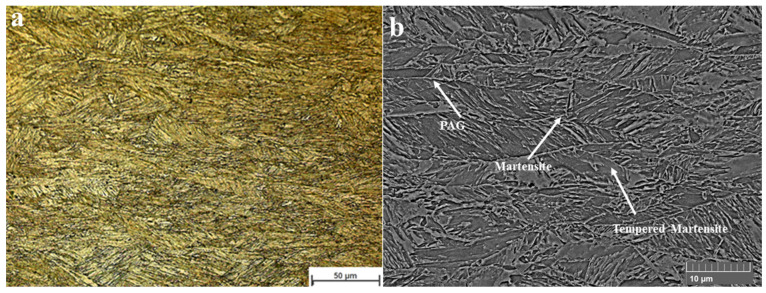
Microstructure of the base material (S1100MC): (**a**) LOM and (**b**) SEM.

**Figure 4:**
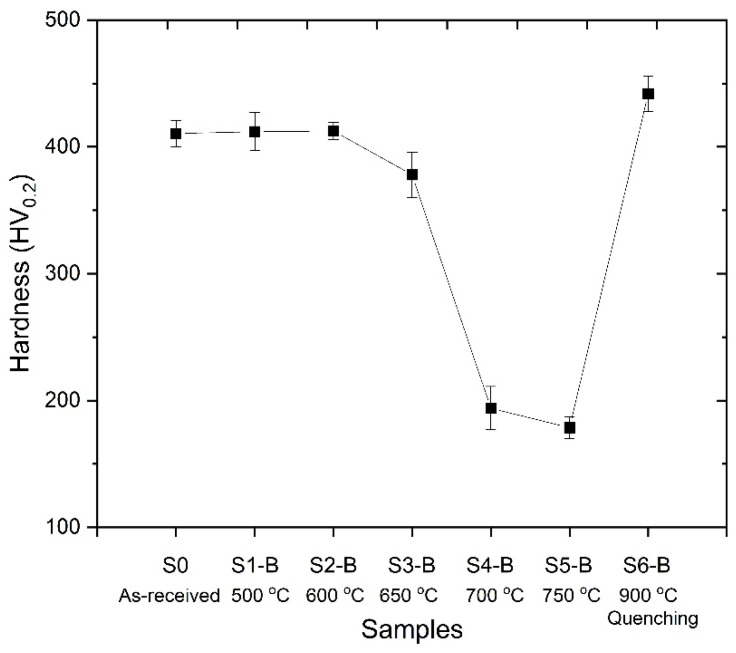
Hardness values of the base material after heat treatment processes.

Figure 3 shows the microstructure of the base material which comprises martensite, tempered martensite and oriented prior austenite grains (PAG). The high strength and toughness values provided in UHSS can be obtained owing to its microstructure. The mixture of the PAG and martensite phases provides enhanced strength and toughness values [20]. The average hardness values of the heat-treated base materials were given in Figure 4. The hardness value of the base material (S0) was determined as 410.5 ± 10.4 HV_0.2_. However, S1-B and S2-B samples have similar hardness values compared to the initial material (S0). Applied heat treatment processes to the base material at 500 °C (S1-B) and 600 °C (S2-B) did not cause any significant change in the hardness. Thermo-Calc analyses also support that there was no phase transformation in the structure at that temperature range.

**Figure 5:**
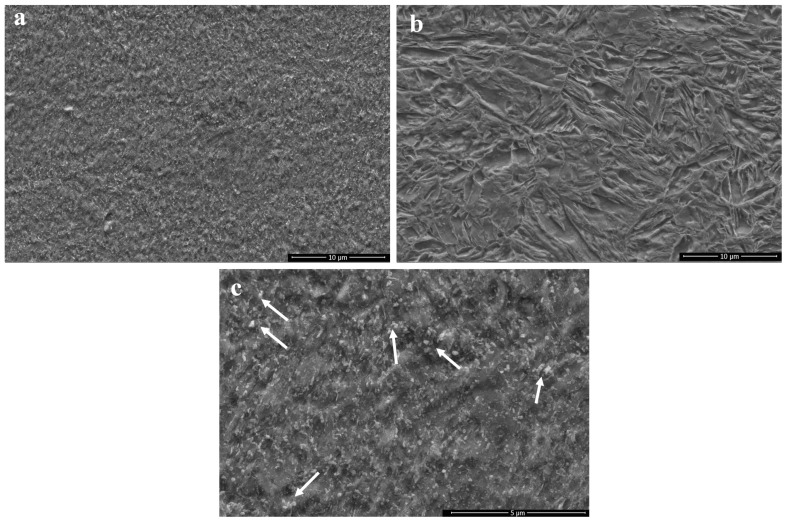
BM microstructure: (**a**) 650 °C/2 h with cooling in the furnace (S3-B), (**b**) 900 °C/5 min. direct quenching (S6-B), (**c**) higher magnification of 650 °C/2 h with cooling in the furnace.

Enhanced heat treatment temperatures caused a decrease in hardness values. Increasing heat treatment temperature led to the breakdown of the tempered martensite and oriented prior austenite grains (PAG). The transformation of the martensite structure resulted in drastic hardness decreases due to the formation of stable phases. Generally, the precipitation of nano-sized alloy carbides in steels can be achieved by tempering martensitic steels, which are supersaturated with carbon and carbide-forming elements—a process analogous to the ageing treatment commonly applied in other metallic materials [21]. Also, Thermo-Calc analyses showed that M_6_C and M_23_C_6_ type carbides precipitate above 550–600 °C, as given in Figure 2. However, despite the evaluation indicating carbide formation, a hardness decrease was observed in samples S3-B, S4-B and S5-B. These hardness decreases can be attributed to grain coarsening owing to increasing heat treatment temperature [22]. Moreover, it was revealed that a reverse martensite to austenite transformation occurs at heat treatment process applied above A_1_ (721 °C) [23]. Accordingly, a drastic hardness decrease was observed in S4-B and S5-B samples due to process temperatures of 700 °C and 750 °C. On the other hand, a significant hardness increase was observed in S6-B compared to other samples. S6-B was annealed at 900 °C for relatively shorter duration and followed by quenching. The higher process temperature and quenching caused the microstructure to form a fresh martensitic structure instead of annealing, resulting in the highest hardness value. Figure 5 shows the microstructures of the S3-B and S6-B samples. The S3-B was cooled in the furnace and the S6-B was quenched following the heat treatment. Accordingly, it was observed that the S3-B sample consisted of tempered microstructure with homogenous carbide distribution. Moreover, it was determined that the carbide coarsening was observed due to the applied process temperature and cooling condition, indicated with white arrows in Figure 5c. In the S6-B sample, a fresh martensite structure was observed. It has been previously stated that the base metal microstructure is dominated by martensite. The heat treatment applied at 900 °C led the formation of austenite compatible with the Thermo-Calc analysis and previous studies [24]. Additionally, rapid cooling conditions provided the formation of fresh martensite.

### 3.3. Characterization of the Weld Metal

**Figure 6:**
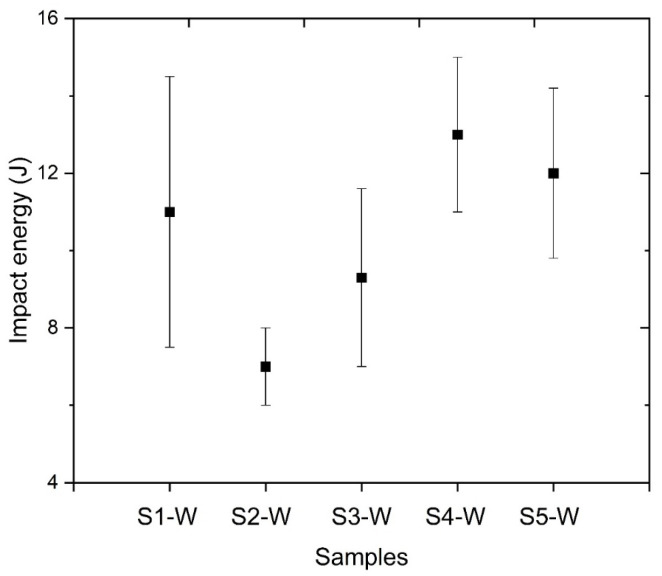
Toughness values of weld metal (WM) after heat treatments.

The Charpy impact test values of the furnace cooled samples were given in Figure 6. In general, a moderate impact toughness with larger scatter/standard deviations was measured for the samples S1-W to S5-W. However, S2-W and S3-W samples have the tendency to lower impact energy values compared to other heat-treated samples (S2-W 7.0 ± 1.0 J and S3-W 9.3 ± 2.3 J). Lee et al. reported that coarsening of the carbides has a critical effect on the toughness of the structure and the steel can be prone to brittle fracture behaviour [25]. As mentioned in the Thermo-Calc analyses and characterization studies of the base material, the temperature range of 600–650 °C is the most effective temperature for carbide coarsening. The coarsening reduced the impact strength, and an embrittlement occurred in S2-W and S3-W samples. The lowest impact energy value was obtained especially in the S2-W sample. In the authors’ previous study [9], the impact tests of FZ did not provide a sufficient toughness value because the material had a high hardness and an untempered martensitic microstructure. The Charpy-V notched impact toughness values for FZ were 20.3 ± 1.6 J, which are below the required value of 27 J at −20 °C, but higher than the values obtained after the heat treatments specified in the present study.

**Figure 7:**
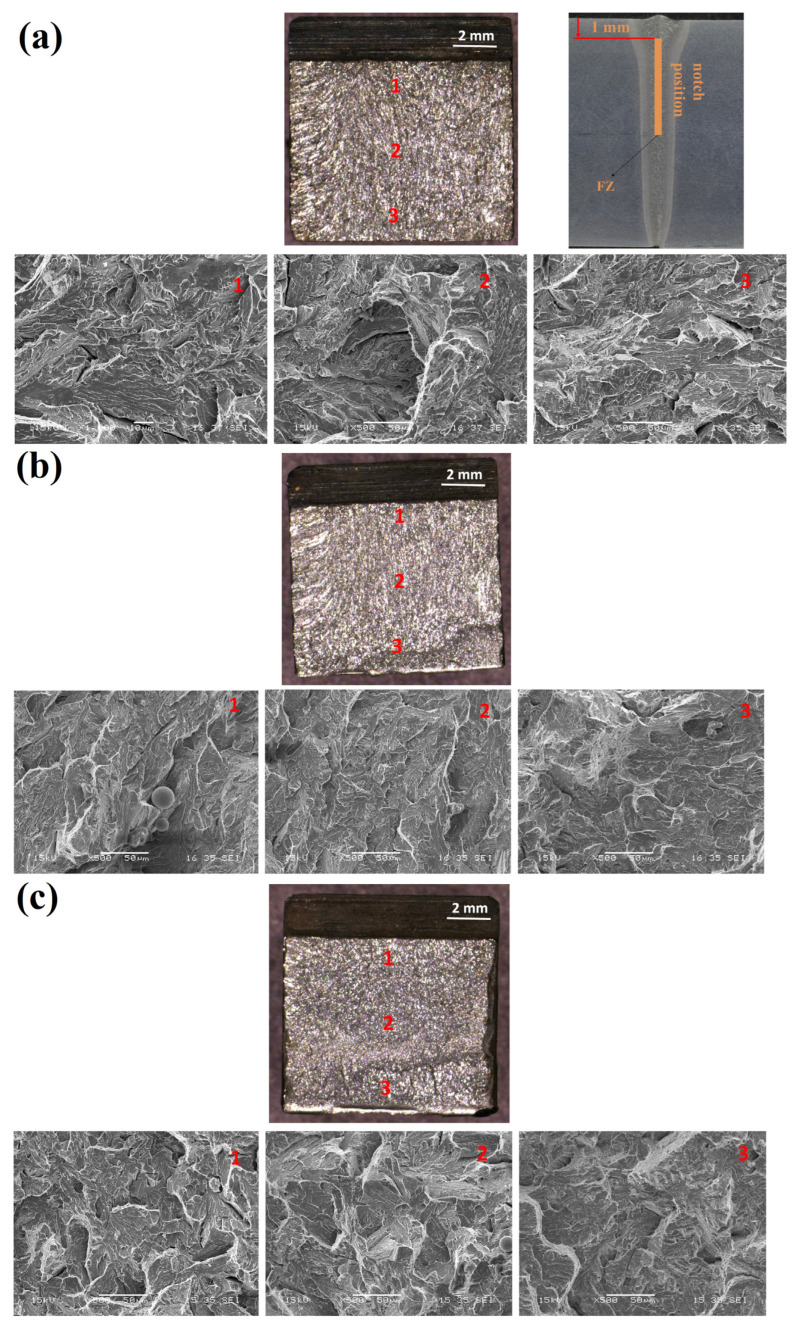
WM fracture surfaces: (**a**) 600 °C/2 h with free cooling in the furnace (S2-W), (**b**) 650 °C/2 h with free cooling in the furnace (S3-W), (**c**) 700 °C/2 h with free cooling in the furnace (S4-W).

The microstructures of the fracture surfaces of the S2-W, S3-W and S4-W samples following the impact tests were given in Figure 7. It was observed that occurrence of plastic deformation was very low, and no lateral expansion was observed on the fracture surface. Accordingly, it was concluded that all specimens showed a brittle fracture. Although the brittle nature was evident in the initial, middle, and end regions of the fractures. The toughness of the weld metal is influenced by several factors like martensite formation, carbide precipitation, and prior austenite grain size [13].

**Figure 8:**
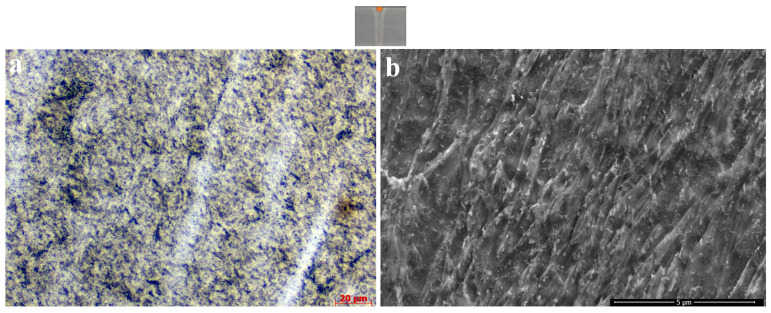
Microstructure of the S3-W sample observed by (**a**) LOM and (**b**) SEM.

The mechanical properties of this UHSS is derived from the combination of fine grain size, solid solution strengthening with additional interstitial hardening, precipitation hardening from carbides, dislocation hardening, and mixed microstructure [26]. In the authors’ previous studies [9,10], the microstructure of the as-received EBW weld metal was discussed in detail. The weld metal microstructure was rapidly cooled due to the nature of the EBW process, resulting in the formation of a completely martensitic structure [9,10]. In the present study, the most important reason for examining this temperature in the heat treatment applied at 650 °C was the partial hardness reduction; the dense carbide formations in the microstructure were remarkable (Figure 8). The most important reason for the decrease in the toughness values of weld metal was the formation and coarsening of carbides in the fusion zone.

**Figure 9:**
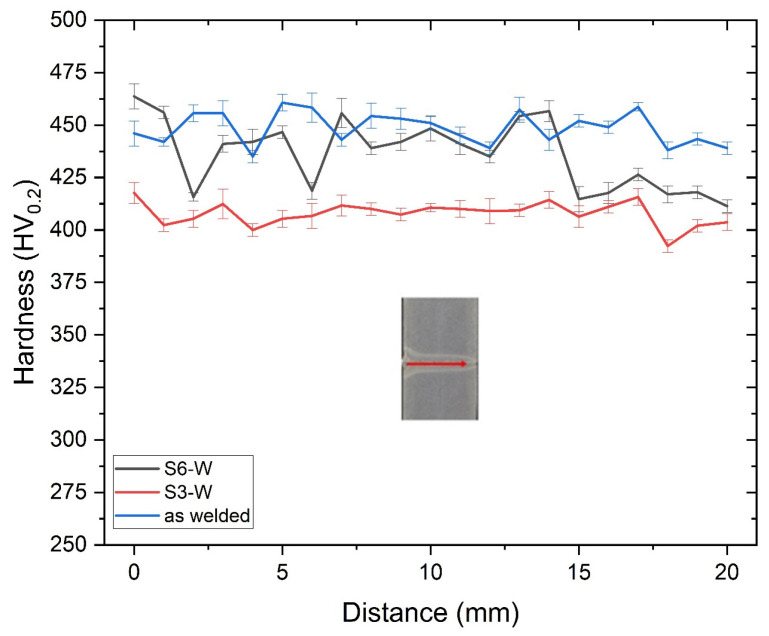
Hardness values of as-welded and S3-W and S6-W samples; red arrow indicates the measuring location and direction.

Heat treatments applied between 500 and 750 °C temperature ranges caused deterioration of the properties in the weld metal structure. The most important reason for the deterioration of the properties was the coarsening of the carbides. Coarse carbide structure had a negative effect especially on toughness properties. Therefore, a new heat treatment procedure was designed. Accordingly, weld metal was held at 900 °C for 5 min and quenched. It was aimed to prevent both carbide precipitation and grain growth with short processing time in the newly designed heat treatment procedure. As a result of that process, the hardness value of the fusion zones (S6-W) was given in Figure 9 in comparison with the hardness value of the S3-W.

**Figure 10:**
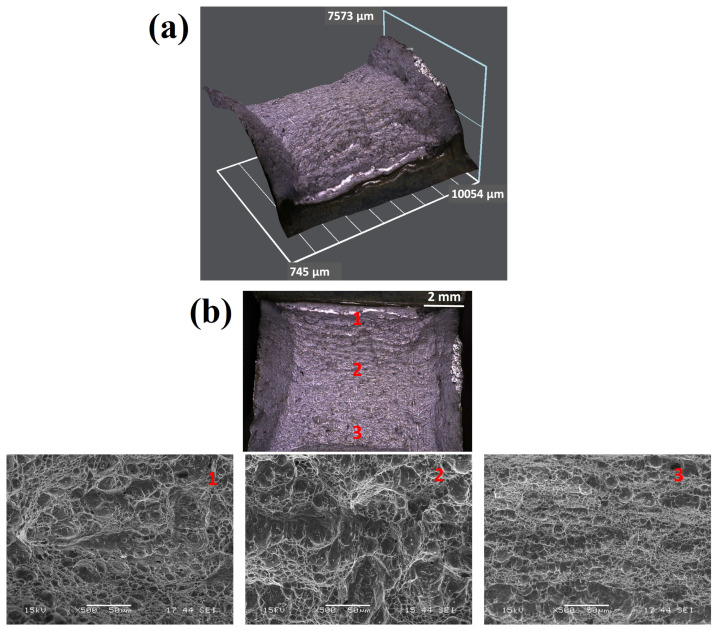
(**a**) Charpy impact test facture surface profile of S6-W, and (**b**) fracture surface SEM images of S6-W.

The average hardness values of the S3-W and S6-W samples were determined as 408 ± 10 HV_0.2_ and 437 ± 20 HV_0.2_, respectively. No significant hardness loss was observed in the S6-W sample following the heat treatment process because the as-welded sample hardness value was 449 ± 12 HV_0.2_. The impact test was carried out at room temperature and −20 °C for S6-W sample. The test results were determined as 136 J and 63 J at room temperature and −20 °C, respectively. As can be seen in the Thermo-Calc analysis, the heat treatment temperature for S6-W prevented the carbide precipitation mechanism and provided sufficient toughness values. On the other hand, the fracture profile and fractographs of the S6-W after the notch impact test applied at room temperature were given in Figure 10. The sample has a significant lateral expansion and ductile network structure. The fracture profile showed a lateral expansion indicating ductile fracture. Moreover, the dimples, which are the typical microstructure of the ductile fracture, were clearly seen in the SEM images [27].

**Figure 11:**
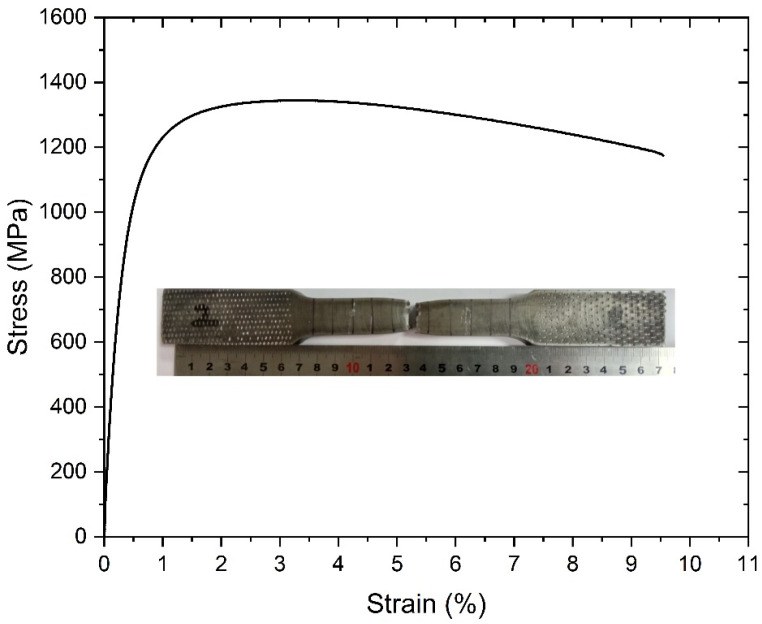
Strain–Stress curve of S6-W sample.

**Figure 12:**
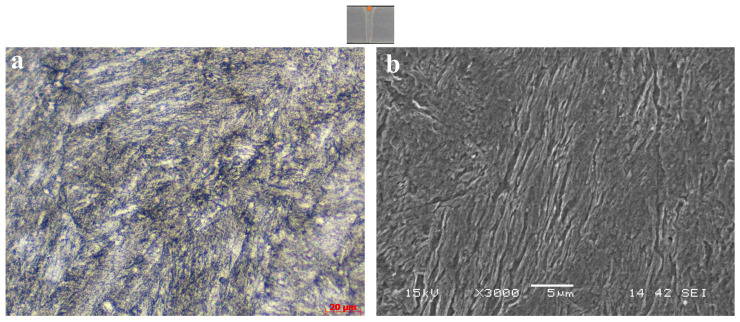
WM microstructure: 900 °C/5 min. with direct quenching observed by (**a**) LOM and (**b**) SEM.

As a result of the heat treatment applied to the S6-W specimen, the desired hardness distribution and toughness values were obtained. Accordingly, a tensile test was performed to determine and control the tensile properties of the S6-W, and the results were given in Figure 11. The rupture occurred at the middle of the sample, which is the fusion zone. Moreover, the yield strength and tensile strength values of the S6-W were determined as 1092 MPa and 1345 MPa, respectively. Additionally, the fracture elongation reached up to 9.4%. Tensile test results also showed that the S6-W specimen acquired the desired mechanical properties. Due to the prevention of carbide precipitation, the S6-W sample provided the desired hardness, strength and toughness values. The microstructure of the S6-W sample can be seen in Figure 12. The microstructure consisted of a chaotic lath martensite pattern.

Coarse carbide structures seen in the microstructure of S3-W sample were not observed in the microstructure of the S6-W sample. In accordance with Thermo-Calc analyses, martensite was formed following the annealing at 900 °C and quenched. Despite the formation of martensite, no significant decrease was obtained in toughness value. In particular, an improved mechanical behaviour was observed compared to carbide-containing samples. Despite the formation of martensite, a significant improvement was achieved in toughness value by eliminating the carbide structure, compatible with Mondiere et al. [28].

## 4. Conclusions

The present study demonstrates that the toughness of UHSS weld metal can be increased by suitable post-weld heat treatment. Furthermore, it was also demonstrated that the requested strength and toughness can be achieved simultaneously in the EBW joint. The issue of low toughness values obtained by using characteristic electron beam welding of UHSS materials can be solved by a post-heat treatment and by obtaining a fresh martensitic microstructure through the rapid water cooling of the joint from the austenite temperature, which is about 900 °C/5 min. It is evident that the precipitation hardening mechanism within the freshly formed martensitic structure is ineffective, leading to a low carbide distribution. In this way, a higher tensile strength of 1345 MPa and an impact toughness of 63 J at −20 °C were achieved, exceeding the required minimum values of 1160 MPa and 27 J at −20 °C of the base material [29].

## Figures and Tables

**Table 1:** Heat treatment procedure.

Sample Code		Temperature	Heating Rate (°C/min)	Holding Time (min)	Cooling
Base Metal	Weld Metal				
S1-B	S1-W	500	15	120	Free cooling in furnace
S2-B	S2-W	600			
S3-B	S3-W	650			
S4-B	S4-W	700			
S5-B	S5-W	750			
S6-B	S6-W	900	30	5	Quenching

## Data Availability

The original contributions presented in this study are included in the article. Further inquiries can be directed to the corresponding author.
